# Sedation and Analgesia for Liver Cancer Percutaneous Radiofrequency Ablation: Fentanyl and Oxycodone Comparison

**DOI:** 10.7150/ijms.47067

**Published:** 2020-08-12

**Authors:** Jiangling Wang, Xiaohong Yuan, Wenjing Guo, Xiaobin Xiang, Qicheng Wu, Man Fang, Wen Zhang, Zewu Ding, Kangjie Xie, Jun Fang, Huidan Zhou, Shuang Fu

**Affiliations:** Department of Anaesthesiology, Cancer Hospital of the University of Chinese Academy and Sciences. Zhejiang, Hangzhou, 310022, China.

**Keywords:** Oxycodone, Fentanyl, Analgesia, Liver cancer radiofrequency ablation

## Abstract

**Background**: Sedation and analgesia use in percutaneous radiofrequency ablation (RFPA) for liver cancer is a necessary part of the procedure; however, the optimal medicine for sedation and analgesia for PRFA remains controversial. The aim of this study was to compare the perioperative pain management, haemodynamic stability and side effects between oxycodone (OXY) and fentanyl (FEN) use in patients under dexmedetomidine sedation.

**Methods:** Two hundred and five adults with an American Society of Anaesthesiologists physical status score of I to II were included in this study. Patients were assigned to the OXY (n=101) or FEN (n=104) group. Radiofrequency ablation was performed under spontaneous breathing and with painless anaesthesia administered intravenously. The outcomes included fluctuations in mean arterial pressure, heart rate, side effects and the perioperative numerical rating scale (NRS).

**Results:** Radiofrequency ablation was successfully performed in 205 patients. No significant differences were observed in mean blood pressure fluctuations between the two groups despite the longer durations of ablation and total sedation time in the OXY group. The highest NRS score during the surgery and 1 hour and 2 hours after the surgery were significantly lower in the OXY group than in the FEN group. Heart rate fluctuations were significantly lower in the OXY group than in FEN group throughout the surgery. More patients in the FEN group displayed unwanted body movement and respiratory depression.

**Conclusions:** Both oxycodone and fentanyl can be applied for liver cancer percutaneous radiofrequency ablation; however, oxycodone provides a better patient experience, lower postoperative pain, less respiratory depression and stable haemodynamic fluctuations.

## Introduction

Liver cancer is the fifth most common cancer worldwide and the third cause of cancer-related death. More than half a million people are diagnosed with hepatocellular carcinoma (HCC) each year globally, over half of whom live in China 1.

Although different treatments, such as surgery, chemotherapy, radiotherapy and liver transplantation, are available for HCC, surgical resection is considered the first-line treatment option for patients with solitary tumours and no portal hypertension. Percutaneous radiofrequency ablation (PRFA) is recommended as the main ablative therapy for local disease of less than 5 cm or tumours not suitable for surgical resection 2-4. Percutaneous radiofrequency ablation is a procedure based on tumour necrosis through local tissue heating via applied an electrode needle in the certain part of the tumour 5, 6. Both general anaesthesia and local anaesthesia are utilized in liver cancer percutaneous radiofrequency 7, 8. Fentanyl (FEN) is a traditional opioid used for pain control and anaesthesia. Studies have also shown that oxycodone (OXY) has comparable effects for pain relief compared with FEN 9-11. Moreover, OXY have a longer half-life compared with fentanyl, and allows evaluation of the pain threshold evaluation, thus providing selective effects on visceral pain 12, 13. As no publications have compared the anaesthetic and analgesic effects between OXY and FEN during liver cancer radiofrequency ablation, this study aimed to determine whether OXY can provide equally effective or superior sedation and analgesia compared with FEN in the radiofrequency ablation of liver cancer when considering haemodynamic stability, pain management and side effects.

## Materials and Methods

### Patients

This study was approved by the Ethics Committee of Zhejiang Cancer Hospital. Written informed consent was obtained from all patients. A total of 210 patients with liver cancer were observed from September 2015 to April 2016, and 205 patients meet the inclusion criteria and were included in the final analysis. Regardless of whether they had primary cancer or metastasis, the patients were first diagnosed with HCC via ultrasound or computed tomography (CT). The participants were randomly assigned to either the OXY group or the FEN group. All patients underwent CT- or ultrasound-guided radiofrequency ablation. The final diagnosis was confirmed by cell pathology after the surgery.

## Methods

The inclusion criteria were as follows: blood pressure (5 minutes after entering the operating room) in the supine position ranging from 90-160/50-100 mmHg, American Society of Anesthesiologists class I-II, Child-Plug score as class A, and no known allergies to the drugs used in the study. The exclusion criteria were as follows: any electrocardiograph (ECG) examination abnormalities, confirmed QTc ECG abnormalities (heart rate-corrected Q and T peak intervals greater than 450 ms) or other cardiac risk factors, such as heart failure, hypokalaemia, or a family history of long QT syndrome; portal hypertension; moderate to severe obstruction or restriction of lung function; or a resting heart rate less than 40 or greater than 100 beats per minute.

The OXY and FEN groups included 101 and 104 patients, respectively. The patients were placed on continuous ECG monitoring after entering the operating room. Both groups received continuous dexmedetomidine (Jiangsu Hengrui Medicine Co., Ltd., Lianyungang, China) infusion (1 µg/kg) for sedation within 15 minutes of the start of the surgery and a maintenance dose of 0.5 µg/kg/h. Local anaesthesia was induced by administering 7 ml of 2% lidocaine before the ablation needle was first inserted into the tumour. Then, 0.15 mg/kg oxycodone (diluted to 1 mg/ml with normal saline; Mundipharma, Vantaa, Finland) was administered to the OXY group, and 2.0 µg/kg fentanyl (diluted to 50 µg/ml with normal saline; Yichang Renfu Inc., Yichang, China) was administered to the FEN (control) group. Both drugs were injected via a peripheral vein. Half the dose of each drug was injected 2 minutes before the position of the tumour was located under ultrasound or CT guidance, with the remaining half administered 2 minutes before the ablation started. Rescue opioids were administered when the numerical rating scale (NRS) score was greater than 4 or when the patient had obvious, unwanted body movements. The rescue OXY dose was 0.05 mg/kg with the total dose not to exceed 0.25 mg/kg, and the rescue FEN dose was 0.05 µg/kg with the total dose not to exceed 3 µg/kg (including the previous dose). Tramadol (Grunenthal Co Ltd, Stolberg, Germany) was administered if opioid administration exceeded the maximum dose. Blood pressure was measured every 5 minutes as well as 2 minutes after the drug was administered or at the discretion of the anaesthesiologist. All patients remained in the recovery room under monitoring for at least 30 minutes, and they were discharged once their Aldrete score was between 9 to 10. Mean blood pressure, heart rate and respiratory rate were recorded at six time points: 5 minutes after entering the operating room (baseline value T1), after the electrode needle was inserted into the liver (T2), at the start of the ablation (T3), the peak value during the ablation (T4), 5 minutes after the end of the operation (T5) and when the patient was discharged from the recovery room (T6). The highest NRS score during surgery (NRS), and the NRS score at 1 hour (NRS1), 2 hours (NRS2) and 6 hours after surgery (NRS6) were recorded for the final analysis. The incidence of nausea, vomiting and side effects such as respiratory depression were also recorded and analysed.

### Statistical analysis

All data were recorded in Excel. SPSS statistical analysis software version 22.0.0.0 (IBM Corp., USA) was used for the data analyses. In previous studies, the postoperatively mean NRS score was 4.3 in FEN group and 3.35 in OXY group. The minimal sample size in this study was 99 patients in each group in order to obtain 90% statistical power and 0.05 α error, under the formula of: 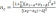 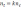. We enrolled 105 patients for each group in this study due to incomplete data collection and exclusion as well as considering that more accurate results would be obtained if additional subjects had been added for the final analysis. Independent-samples *t* tests were used to evaluate normally distributed continuous variables. Non-normally distributed variables were compared using the Mann-Whitney U test. Categorical variables were compared using the chi-squared or Fisher's exact tests. *P*<0.05 was considered to indicate significance for each hypothesis.

## Results

**Figure 1:**
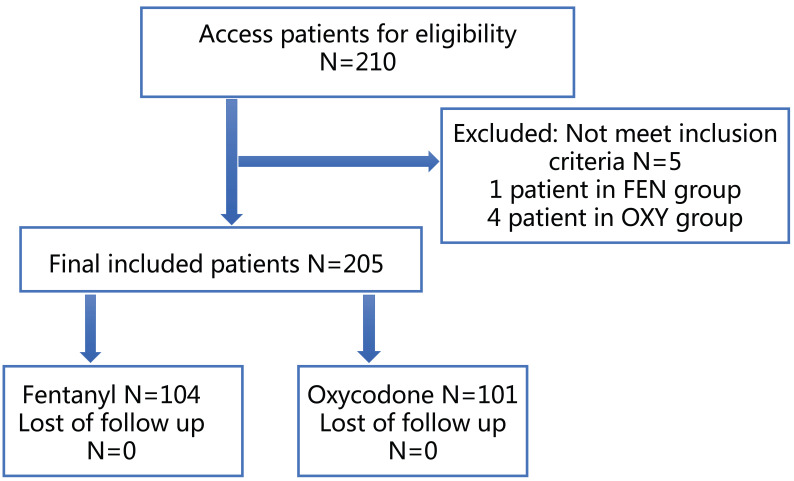
Flowchart of the study design and patients included.

A total of 210 patients were included in this study, of which 4 patients in the OXY group and 1 patient in the FEN group did not meet the inclusion criteria; therefore, 101 patients in the OXY group and 104 patients in the FEN group were included in the final analysis (**Figure 1**). The baseline factors of the two groups are shown in **Table 1.** No significant differences in the basic characteristics were found between the two groups. The operative duration was longer in the OXY group than in the FEN group.

**Figure 2:**
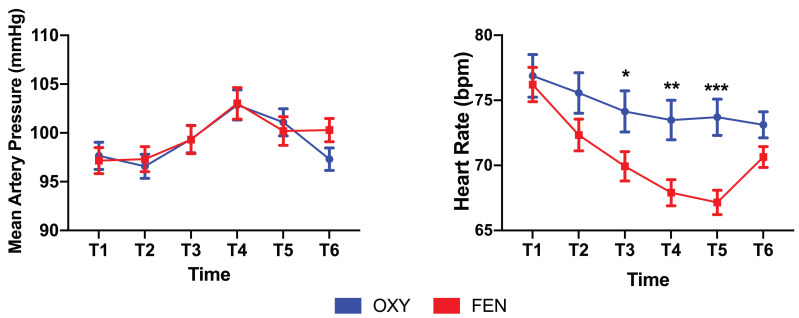
Fluctuations of mean artery pressure (MAP) and heart rate at indicated time points perioperatively. MAP was not significantly different between two groups. Heart rates were significantly lower in fentanyl group, especially during period after the ablation begins. The lower heart rates even persist after the procedure finished and recovered before discharge to the ward. Data were presented as the mean ± SD and compared by using two-sample Student's *t*-test. *P, 0.05, **P, 0.01 and *** *P,* 0.001. **Abbreviations:** OXY, oxycodone; FEN, fentanyl.

**Figure 3:**
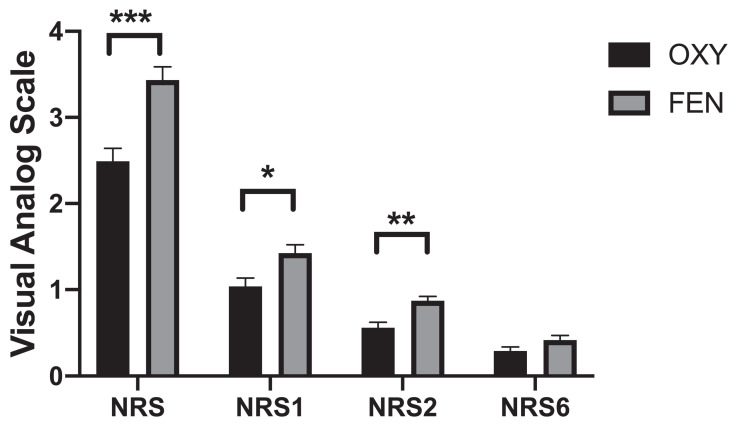
Highest NRS score during surgery and NRS score at indicated time points postoperatively between two groups. Data were presented as the mean ± SD and compared by using two-sample Student's *t*-test. *P, 0.05, **P, 0.01 and *** *P,* 0.001. **Abbreviations:** OXY, oxycodone; FEN, fentanyl.

No significant differences were found in terms of mean arterial pressure fluctuations between the two groups; however, mean heart rate fluctuations were significantly different between the two groups. The heart rates were significantly lower at the T3 (74.1±15.9 VS 69.9±11.5, *p*=0.03), T4 (73.5±15.2 VS 67.9±10.2, *p*=0.002) and T5 (73.7±15.2 VS 67.2±9.6, *p*=0.000) time points in the FEN group than in the OXY group (**Figure 2**). The OXY and FEN groups had 9 (≥50% of the initial value or ≥180/100 mmHg) and 10 cases of hypertension during the surgery, respectively. No cases of hypotension were noted in either group (≤50% of the initial value or ≤80/40 mmHg). The perioperative highest NRS score (2.49±1.56 vs 3.41±1.52, *p*=0.000), NRS1 (1.06±0.99 vs 1.39±0.96, *p*=0.015) and NRS2 (0.56±0.67 vs 0.86±0.53, *p*=0.001) were significantly lower in the OXY group than in the FEN group (**Figure 3**). More patients in the FEN group displayed unwanted body movements (6.9% VS 15.4%, *p*=0.044) and respiratory depression (1% VS 12%, *p*=0.002). The NRS6 score, the number of patients receiving rescue anaesthetic and the incidence of nausea and vomiting were not significantly different between the two groups (**Table 2**). One case of slight respiratory depression (respiratory rate between 8-10 breaths/min or an SPO_2_ drop of 3-6% during 6 L/min oxygen inhalation) occurred in the OXY group; however, 6 cases of moderate respiratory depression occurred in the FEN group (respiratory rate ≤8 breaths/min or an SPO_2_ drop of more than 6% during 6 L/min oxygen inhalation), and the remaining 6 cases were mild.

## Discussion

In summary, the use of oxycodone in PRFA procedures results in lower perioperative NRS scores, fewer side effects and more stable haemodynamic fluctuations than the traditional opioid FEN.

PRFA is a safe and local effective treatment for liver carcinomas that is less invasive than other procedures 14, 15. PRFA causes pain and patients are more likely to experience severe pain when the tumours are close to the liver capsule, phrenic vein, large vessels or the gallbladder 16. Fentanyl is 50 to 100 times more potent than morphine in pain management 17. While OXY was proved to have equally effective and equipotent in postoperative pain management compared with morphine 18, 19. OXY also has significant effects in pain-relieving effects on malignant and non-malignant pain 20. A previous study showed that dexmedetomidine can be infused by a continuous intravenous drip at a concentration range of 0.5-2 µg/kg/h, which can provide acceptable levels of sedation, and that dexmedetomidine plus FEN can provide satisfactory sedation and analgesia as well as postoperative analgesia for outpatient dental extractions performed under local anaesthesia 21. In this study, a 1 µg/Kg loading dose and 0.5 µg/kg/h maintenance dose of dexmedetomidine, 0.15 mg/kg of oxycodone and 2 µg/Kg of fentanyl were used. Results showed they have same sedation and analgesia effects perioperatively.

All patients in both groups underwent the PRFA procedure. The duration of both sedation and ablation were both longer in the OXY group, possibly due to the higher number cases of CT guided ablation. Usually, more time is needed for CT guided when positioning. The perioperative NRS scores seemed to have statistically significant differences between two groups in this study, but the clinically significance remain controversial as NRS scores in both groups were low during the postoperative period even through the NRS scores in OXY group were lower than those in FEN group at all time points. Different from FEN, a µ-opioid receptor opioid, OXY acting on µ and κ receptors and has been shown as an effective analgesic for postoperative pain management after abdominal surgery with severe visceral pain 22, 23. One of possible mechanisms for treating visceral pain is the activation of sodium channels expressed on gut afferent nerves by peripheral κ receptor agonists 24, 25. The lower NRS scores in OXY group during perioperative period may due to the activation of κ opioid receptor.

With conscious sedation or the use of any other analgesic the most important consideration is airway protection 26, especially when this procedure is undergoing outside operating room. Respiratory depression is mainly associated with µ and δ opioid receptors. Activation of µ opioid receptors in the central nervous system are associated with adverse effects such as sedation, respiratory depression and nausea 27. Fentanyl can affect both the onset and offset of breathing and cause respiratory depression 28, while κ receptor agonists did not induce respiratory depression 25. There were 13 cases of respiratory depression in this study with 12 in the FEN group and 1 in the OXY group, which remains consistent with the previous conclusions. Thus, this study has provided another evidence that OXY may be safer in PRFA when considering the side effects such as respiratory depression and hemodynamic stability as both FEN and OXY have same sedation and analgesic effects during these procedures.

No significant differences were observed in blood pressure fluctuations between the two groups; however, the heart rate dropped significantly lower in the FEN group during the ablation. The mechanisms of this effect are currently unclear. One possible reason is that OXY may act on the Ach-IKACh pathway and IKACh is specifically expressed in atrial myocytes 29. Studies have demonstrated that OXY mainly acts on Kir3.1 channels at supraspinal sites and in the brain 30. Whether OXY acts on these channels in atrial myocytes requires future analysis.

Our study has some limitations that should be noted. First, patients with hepatitis-related dysfunction, such as aminotransferase and bilirubin abnormalities, were included, which may have increased the selection bias of the study. Second, this study had a small sample size, and further clinical studies using these anaesthetics are needed to confirm the conclusions. Third, being a single centre and small number randomized control trial (RCT), further RCTs in multiple centres with more patients are needed to confirm our study's conclusions. Fourth, the sample size was larger than the results from the power analysis. We enrolled 105 patients for each group in this study due to incomplete data collection and exclusion as well as considering that more accurate results would be obtained if additional subjects had been added for the final analysis. Finally, being a clinical study, further studies are needed to confirm the underlying mechanisms of the heart rate decrease and respiratory depression in the FEN group.

## Conclusions

Use OXY for liver cancer percutaneous radiofrequency ablation led to a reduced respiratory depression and heart rate decrease during these procedures.

## Figures and Tables

**Table 1:** Baseline factors and perioperative variables of two groups

Characteristics	OXY (%) n=101	FEN (%) n=104	*P* value
Age-years (±SD)	57.9±9.9	58.2±10.5	0.823
Weight (Kg)	61.8±9.1	61.1±9.3	0.562
Gender, Male (n%)	80 (79.2)	73 (70.2)	0.138
Cause of Tumor, primary cancer (n%)	57 (56.4%)	52 (50.0)	0.217
Tumor size (±SD)	2.9±1.7	2.6±1.3	0.12
Alcohol (n%)	52 (51.5)	47 (45.2)	0.367
Hypertension (n%)	26 (25.7)	25 (24)	0.778
Diabetes (n%)	14 (13.9)	8 (7.7)	0.154
Dexmedetomidine (±SD)	85.4±13.4	84.9±13.4	0.813
**Tumor Location**			
The center part of the liver (n%)	90 (89.1)	86 (82.7)	0.13
Subcapsular (n%)	6 (5.9)	11 (10.6)	0.171
Subphrenic (n%)	5 (5.0)	9 (8.7)	0.220
Perivascular or perigallbladder (n%)	3 (3.0)	8 (7.7)	0.214
**Ablation tumor numbers (n%)**			
1	66 (65.3)	66 (63.5)	0.446
2	20 (19.8)	22 (21.2)	0.474
3	15 (14.9)	15 (14.4)	0.544
CT guided (n%)	31 (30.7)	27 (26.0)	0.452
Sedation Time (±SD)	55.29±18.52	47.12±17.70	0.001*
Total Ablation Time (±SD)	17.9±9.4	15.1±9.7	0.036*

Two-sample Student's t-test was used for continuous data and Pearson's chi-squared or Fisher's exact test were used for categorical data. *P<0.05; **Abbreviations:** CT Computed Tomography.

**Table 2:** Differences of NRS scores and intraoperative variables between two groups

Variables	OXY (n=101)	FEN (n=104)	*P* value
Intraoperative highest NRS (±SD)	2.49±1.56	3.41±1.52	0.000*
NRS1 (±SD)	1.06±0.99	1.39±0.96	0.015*
NRS2 (±SD)	0.56±0.67	0.86±0.53	0.001*
NRS6 (±SD)	0.29±0.48	0.40±0.53	0.132
Unwanted body movement ( n%)	7 (6.9)	16 (15.4)	0.044*
**Intraoperative rescue anesthetic (n%)**			0.556
Once (n%)	8 (7.9)	10 (9.6)	
Twice (n%)	3 (3.0)	5 (4.8)	
Nausea (n%)	3 (3.0)	1 (1.0)	0.299
Vomitting (n%)	0	0	-
**Respiratory depression (n%)**			0.002*
Mild	1 (1.0)	6 (5.8)	
Moderate	0	6 (5.8)	
Hypertension (n%)	9 (8.9)	10 (9.6)	0.527
Hypotension (n%)	0	0	-

Intraoperative highest numerical rating scale (NRS) and postoperative NRS scores at different time point in two groups. *P<0.05. Two-sample Mann-Whitney U rank-sum test was used for NRS scores and Pearson's chi-squared test was used for other variables; **Abbreviations:** OXY Oxycodone, FEN Fentanyl, NRS1 Numerical rating scale of 1 hour after surgery, NRS2 Numerical rating scale of 2 hours after surgery, NRS6 Numerical rating scale of 6 hours after surgery.
